# Electrosprayed minocycline hydrochloride-loaded microsphere/SAIB hybrid depot for periodontitis treatment

**DOI:** 10.1080/10717544.2021.1902020

**Published:** 2021-03-27

**Authors:** Ting Zhang, Yingqian Qiu, Jinlin Song, Pengfei Zhou, Hang Liao, Yuting Cheng, Xiaohong Wu

**Affiliations:** a Stomatological Hospital of Chongqing Medical University, Chongqing, China; b Chongqing Key Laboratory of Oral Diseases and Biomedical Sciences, Chongqing, China; c Chongqing Municipal Key Laboratory of Oral Biomedical Engineering of Higher Education, Chongqing, China

**Keywords:** Periodontitis, minocycline hydrochloride, sucrose acetate isobutyrate, drug release, osteogenesis

## Abstract

Minocycline hydrochloride (MINO) has been one of the most frequently used antibiotics in the treatment of periodontitis due to its antibacterial activity and osteogenesis effects; however, high levels of MINO administered during the treatment halt the formation of new bone. Therefore, the purpose of the present study was to prepare a MINO-microsphere/sucrose acetate isobutyrate (SAIB) hybrid depot to reduce the burst release of MINO and ensure antibacterial and osteogenesis effects of MINO in the treatment of periodontitis. Uniform microspheres, approximately 5 µm size, with a slightly rough surface and different MINO loading (10, 12, and 14%) were prepared, and the microspheres were added into SAIB, after which the burst release significantly decreased from 66.18 to 2.92%, from 71.82 to 3.82%, and from 73.35 to 4.45%, respectively, and the release from all the MINO-microspheres/SAIB hybrid depots lasted for 77 days. In addition, cytotoxicity test showed that the MINO-microsphere with 12% drug loading promoted the proliferation of osteoblasts the most and was subsequently used in vivo experiments. Moreover, in the model of ligatured-induced periodontitis in SD rats, the MINO-microsphere/SAIB hybrid depot not only significantly increased the alveolar bone height and bone volume but also reduced the inflammation of the periodontal tissue. Additionally, it also inhibited the expression of the receptor activator of nuclear factor-kappa B ligand (RANKL) and promoted the expression of osteoprotegerin (OPG).. These results indicated that the MINO-microsphere/SAIB hybrid depot might be promising in the treatment of periodontitis.

## 1. Introduction

Periodontal disease, a chronic inflammatory disease of the periodontium, often results in progressive damage of the surrounding alveolar bone (Mou et al., 2019; Munasur et al., 2020). This disease is not only the major cause of tooth loss in adults but also one of the two major menaces to the oral health (Nazir, 2017). The standard treatment for periodontitis is scaling and root planning (SRP); however, the success of SRP mainly depends on clinical skills and it cannot completely remove the bacteria that dwell deep in the periodontal pocket (Do et al., 2015; Nazir, 2017; Mou et al., 2019). Therefore, antibiotics are often combined with SRP to treat periodontitis (Pang et al., 2014).

Minocycline hydrochloride (MINO), a semi-synthetic derivative tetracycline, has broader spectrum of antibacterial activity than other tetracycline antibiotics and has been frequently used to treat periodontal disease (Oliveira et al., 2006; Kashi et al., 2012). In addition to its antibacterial activity, MINO also exhibits pharmacological properties that are effective for the management of periodontitis (Nagasawa et al., 2011). MINO has been proven to restrain bone resorption and promote new bone formation. Pedro Sousa Gomes (Gomes & Fernandes, 2007) demonstrated that 1 µg/ml of MINO significantly improved the proliferation of human bone marrow osteoblastic cells. Furthermore, in our previous study, we demonstrated that appropriate concentration of MINO can upregulate the expression levels of Runt-related transcription factor 2 (Runx2), alkaline phosphatase (ALP), and osteopontin (OPN) in osteoblasts and increase the differentiation and mineralization of the osteoblasts of SD rats (Shao et al., 2018). However, high concentrations of MINO in the periodontal pockets may cause damage to the viable cells of the supportive periodontal tissue, especially the bone formation cells (osteoblasts) (Almazin et al., 2009). Salah M (Almazin et al., 2009) reported the harmful effects of MINO at a concentration of 0.5 mg/mL on osteoblast proliferation in vitro. Pedro Sousa Gomes (Gomes & Fernandes, 2007) also reported that high levels of MINO led to a dose-dependent deleterious influence on osteoblasts and delayed proliferation and differentiation. Therefore, recent studies have focused on local application associated with the minimal inhibitory concentration of MINO and a sustained-release device in order to avoid these adverse effects caused by high local concentration of antibiotics (Vandekerckhove et al., 1998). Nevertheless, the minocycline-loaded poly lactic-co-glycolic acid (PLGA) electrospun membrane that we had fabricated in our previous study had an obvious burst release (up to 20%) on the first day (Ma et al., 2020), thus a carrier loaded with MINO, which aims to further reduce burst release, should be explored in future research.

Among the available sustained-release delivery systems, sucrose acetate isobutyrate (SAIB) is one of the most prospective systems with biodegradability and injectability, and it is usually considered safe by the U.S. Food and Drug Administration (FDA) (Wang et al., 2018; Harloff-Helleberg et al., 2019). Furthermore, the viscosity of the SAIB can be dramatically reduced when mixing with a little amount of solvent, for example, ethanol, allowing the SAIB to be easily inject using small needles. Upon injection, the solvent diffuses from the depot into body fluid, resulting in a highly viscous SAIB depot from where the drug can be released in a sustained manner (Wang et al., 2018; Harloff-Helleberg et al., 2019; Yang et al., 2019). However, burst release still existed in the application of SAIB (Park & Lee, 2009). Xia Lin’s study and our previous study showed that the combination of microsphere and SAIB could significantly decrease burst release of the investigated drugs (Lin et al., 2015; Yang et al., 2019). There are several methods to prepare microspheres, among which electrospray is a promising method with an extremely strict control on size distribution and high encapsulation efficiency for hydrophobic and hydrophilic drugs (Park & Lee, 2009; Furtmann et al., 2017). PLGA is approved by the FDA and has good biocompatibility and biodegradability, and the kinetics of drug release can be precisely controlled from days to months (Ford Versy et al., 2013; Zhang et al., 2014; Gu et al., 2016). Moreover, polyethylene glycol (PEG), as a hydrophilic material, was used to improve the monodispersity and encapsulation efficiency of the electrosprayed microspheres (Dan et al., 2020).

In this study, we prepared MINO-loaded PLGA/PEG microspheres through the electrospray technique with an aim to evaluate their characteristics, such as morphology, size distribution, surface wettability, drug release, and drug degradation. MINO-loaded microspheres (MINO-microspheres) were mixed with SAIB (MINO-microsphere/SAIB; MINO-M-SAIB) in order to attain a slow and continuous release, and the alveolar bone augmentation potential of the fabricated MINO-M-SAIB hybrid depot was studied in SD rats with ligature-induced experimental periodontitis.

## 2. Methods and materials

### 2.1. Materials

PLGA (copolymer ratio 75:25, molecular weight 66,000–107,000) was purchased from Jinan Daigang Biomaterial Co. Ltd. (Shandong, China). Minocycline hydrochloride (MINO), Polyethylene glycol (PEG, Mn = 6 kDa), sucrose acetate isobutyrate (SAIB, density of 1.146 g/mL at 25 °C, MW = 846.91 g/mol) were purchased from Sigma-Aldrich (St. Louis, MO), and the MINO ointment (Periocline^®^) was procured from Sunstar Inc. (Osaka, Japan). For the cell culture of osteoblasts, alpha-modified Eagle’s medium (a-MEM, HyClone), antibiotics (Sigma), and fetal bovine serum (FBS, Gibco, Australia) were used. SD rats were obtained from the animal center of Chongqing Medical University, anti-osteoprotegerin Rabbit pAb (GB11151) and anti-RANKL Rabbit pAb (GB11235) were purchased from Servicebo (Wuhai, China). All chemicals used were of analytically pure grade.

### 2.2. Preparation of MINO-microspheres

PLGA was dissolved in chloroform to form a solution at a concentration of 0.07 g/mL and PEG (5% w/w of PLGA) was added to it to prepare a PLGA/PEG solution. Then, different concentrations of MINO (0%, 10%, 12%, and 14% w/w relative to PLGA) were mixed with the PLGA/PEG solution. The resultant solutions were magnetically stirred at the room temperature for 2 h to obtain complete the dissolution. Then, during electrospraying using a single-nozzle electrospinning setup (Beijing Yongkang Leye Technology Development Co. Ltd., China), the solutions were contained in 5-mL syringes with 20-G needles and pushed at a steady speed of 0.9 ml/h to produce PLGA/PEG microspheres. Meanwhile, a voltage of 14 KV was applied between the needle and the aluminum foil (as the collector of microspheres), and the distance between the electrospraying needle and the aluminum foil was kept at 20 cm. Moreover, the relative humidity and temperature were strictly controlled at 30–35% and 20–25 °C, respectively. Finally, the collectors were placed in an incubator at 37 °C for 2 days to remove the residual solvent. Finally, the dry microspheres were refrigerated at −20 °C for further analysis.

### 2.3. Characterization of MINO-microspheres

#### 2.3.1. Particle morphology

Scanning electron microscopy (SEM; S-3000N, HITACHI, Japan) was used to analyze the morphology and size of MINO-microspheres at an accelerating voltage of 5 kV after gold coating. Moreover, the average diameters of the MINO-microspheres were measured by the ImageJ 2.0 analysis software. The coefficient of variation (CV) was used to assess the monodispersity of the MINO-microspheres and was calculated using the formula (1), meanwhile, the monodispersity of the microspheres increased with a decrease in the CV value.

(1) $$\text{CV}(\%)=\frac{\text{Standard}\;\text{deviation}}{\text{Mean}\;\text{particle}\;\text{size}}*100\%$$

#### 2.3.2. Drug-encapsulation efficacy (EE) and drug-loading efficiency (LE)

The drug EE and LE experiments were conducted in accordance to the ultracentrifugation technique described in our previous study (Yang et al., 2019). The EE represented the amount of MINO entrapped in the microspheres; therefore, 3 mg of MINO-microspheres were dissolved in 1 mL of phosphate-buffered saline (PBS) and blended completely for 5 min to obtain a sample solution; the sample solution was then centrifuged (13000 rpm for 10 min) in a 10-K ultracentrifuge tube in order to deposit the free MINO on the surface of the microspheres. The supernatants were carefully extracted and analyzed at a wavelength of 350 nm using a UV-Vis spectrophotometer (ND-2000; Thermo Scientific). Similarly, the drug LE represented the total amount of MINO in the microspheres, which was determined by dissolving 3 mg of microspheres in 1 mL of absolute ethanol and sonicated for 10 min. The sample solution was allowed to stand until complete dissolution of the polymers was achieved, followed by centrifugation at 15000 rpm for 20 min. The supernatant was measured as described earlier. All experiments were performed in triplicate. The EE and LE of MINO were calculated using formulas (2) and (3), respectively:

(2) $$\text{EE}=\left(1-\frac{\text{free}\;\text{MINO}}{\text{the amount of MINO in the microspheres}}\right)*100\%$$

(3) $$\text{LE}=\left(\frac{\text{the amount of MINO}\;\text{in the microspheres}}{\text{the weight of microspheres}}\right)*100\%$$

#### 2.3.3. Contact angle measurement

The contact angle was measured by using a video constant angle device (VCA Optima, AST Inc.), and the aluminum foil collecting the microspheres was placed on the testing position and covered with a drop of deionized water (approximately 2 μL each time), followed by recording of the value of contact angle immediately. Each group included three samples, and the contact angle of each sample was calculated by determining three different locations.

#### 2.3.4. Laser scanning confocal microscopy

Under certain specific conditions, minocycline can emit yellow-green fluorescence (Dodiuk-Gad et al., 2006). Consequently, the samples of electrosprayed microspheres collected on glass slide were observed under a laser-scanning confocal microscope (TCS SP8X; Leica, Wetzlar, Germany) at an excitation wavelength of 375 nm.

#### 2.3.5. Differential scanning calorimetry (DSC)

DSC measurements were carried out using a DSC-Q2000 (TA Instruments). Accurately weighed 10-mg samples were placed in aluminum pans, and then sealed with an aluminum lid. A sealed empty pan was used as a reference. The heating rate was set to 10 °C/min from 30 °C to 210 °C under a dry nitrogen atmosphere (20 ml·min^–1^).

### 2.4. Preparation of minocycline hydrochloride/SAIB (MINO-SAIB) depot and minocycline hydrochloride-microsphere/SAIB (MINO-M-SAIB) hybrid depots

SAIB was added to ethanol to form a transparent SAIB/ethanol (80/20, w/w) solution. Next, 1 mg of MINO was dispersed into SAIB/ethanol (80/20, w/w) system to prepare the minocycline depot. Equally, microspheres with three different drug loading were respectively dispersed into the SAIB/ethanol (80/20, w/w) solution through eddying for 5 min in order to obtain minocycline hybrid depots. The final drug loading of the prepared MINO-SAIB and MINO-M-SAIB depots were all 20 mg/g.

### 2.5. *In vitro* release

Approximately 50 mg of the MINO-SAIB and MINO-M-SAIB depots (all containing 1 mg MINO) were injected into a 1.5-ml EP tube containing 1 mL of the release buffer (PBS solution, pH 7.4, 0.02% NaN3). In equal concentration, MINO-microspheres (containing 1 mg MINO) were dispersed into the PBS solution. All specimens were placed in the ZWY-110 × 30 reciprocal shaking water bath (Zhicheng Inc, China) at 37 °C. At each predetermined time point, the release buffer was collected by centrifuging (13,000 rpm for 10 min) and replaced with 1 mL of fresh PBS solution. Then, the release buffer solution was analyzed by UV-Vis spectrophotometer at a wavelength of 350 nm. At least three repeats for each sample group were conducted for all experiments.

### 2.6. *In vitro* degradation rate

The *in vitro* degradation rate was determined by measuring the weight loss of microspheres. Then, 10 mg of microspheres were immersed in 1 mL of PBS and preserved in an incubator for up to 90 days at a constant temperature of 37 °C. At each time point (7, 15, 30, 45, 60, 75, and 90 days), the samples were removed from the medium, washed by distilled water, and then dried in an incubator at 37 °C for 2 days. Finally, the degradation rate was calculated using the Equation (5), as follows:

(5) $$\text{Degradation}\;\text{rate}(\%)=\frac{W0-\text{Wt}}{W0}*100\%$$

where, W_0_ is the initial weight of microspheres, W_t_ is the dried weights of microspheres at time = t. Each sample group was tested thrice at least.

### 2.7. The porosity of depots

When the SAIB solution was injected into the aqueous release medium, the solvent in the system diffused into the water phase, while the water phase diffused into the interior of the depot, which led to the formation of some water-rich micropores in the depot. Therefore, the porosity was reflected by measuring the volume of water diffused into the depot. Briefly, approximately 50 mg of the analyzed specimens were injected into 1 mL of release buffer (PBS solution) and placed on a shaker water bath at 37 °C; moreover, at these time points (0, 2, 8, 15, 30, and 45 days), the release buffer was completely removed, after which the absorbed water was removed by lyophilizing the depot. The volume of water absorbed by the depot was calculated by measuring the weight difference in the depot before and after lyophilization. Thus, the porosity was calculated by using formula (7) as shown below:

(7) $$P=\frac{(W2-W3)/\rho 1}{W1\times C/\rho 2+(W2-W3)/\rho 1}*100\%$$

where, p is the porosity; W_1_, W_2_, and W_3_ are the initial weight of SAIB solution; and the weight of depot absorbing water and the weight of lyophilized depot, respectively. ρ^1^ and ρ^2^ are the density of water and of SAIB, respectively. C is the concentration of SAIB in the SAIB solution. Each sample group was repeated at least thrice for all experiments.

### 2.8. Fourier transform infrared spectroscopy (FTIR)

FTIR (Thermo Scientific Nicolet iS5) was used to analyze the chemical composition of the MINO-microspheres, MINO-M-SAIB hybrid depot and their compositions, and the wave number range was 500–4000 cm^−1^. The MINO, PEG, PLGA, MINO-microspheres, and MINO-M-SAIB hybrid depot samples were analyzed by attenuated total reflection (ATR), while the SAIB was analyzed by the transmission method.

### 2.9. Cytotoxicity of the drug delivery systems on osteoblastic cells

#### 2.9.1. Cell culture

SD rats (age: 2–3 days) were killed through cervical dislocation and then sterilized in 75% alcohol for 5 min, after which their cranial bones were collected and cultured for several days to obtain the osteoblast cells. The osteoblast cells were next cultured in a-MEM with 10% FBS and 100 U/mL antibiotics (penicillin-streptomycin-amphotericin) at 37 °C under 5% CO_2_ atmosphere. The culture medium was replaced every second day.

#### 2.9.2. Cck-8 assay

The cytotoxicity of the MINO-microspheres, MINO-M-SAIB and MINO-SAIB depots was assessed by the CCK-8 assay. The MINO-microspheres, MINO-SAIB and MINO-M-SAIB depots (all containing 1 mg MINO) were respectively immersed in 1 ml of culture medium (containing a-MEM with 10% FBS and 100 U/mL antibiotics) and then placed in 37 °C incubator for 24 h to obtain their respective extracts. Meanwhile, the extracts were degermed through a 22-μm filter and then refrigerated at 4 °C for subsequent experiments. The osteoblast cells were seeded at a density of 2 × 10^4^ cells/well in a 96-well plate. After 24 h of cell adhesion, the old medium was discarded and 100 μL of different extracts were added to treat the cells. Next, 100 μL of the culture medium (containing a-MEM with 10% FBS and 100 U/mL antibiotics) was used as the control. After 24 h, the cell culture medium was removed and substituted with 100 μL of fresh culture medium and 10 μL of CCK-8. After 3 h, the OD values were measured by an enzyme-linked immunosorbent assay (ELISA) plate reader at a wavelength of 450 nm (Bio-Tek, Winooski, VT).

### 2.10. Animal experiments

#### 2.10.1. Establishment of periodontitis

The experimental procedures were approved by the Ethics Committee of the Affiliated Stomatological Hospital of Chongqing Medical University (Approval no. [2019] 34), and 7-week-old female Sprague–Dawley rats (from the Institute of Experimental Animal Center of Chongqing Medical University) were randomly assigned into five groups (*n* = 5): (1) Control (no ligation), (2) ligation, (3) ligation + M-SAIB, (4) ligation + MINO-M-SAIB, and (5) ligation + Periocline^®^. In order to establish a periodontitis model, 10% chloral hydrate (4 mL/kg) was used to anesthetize the experimental rats via intraperitoneal injection and a 0.2-mm-diameter orthodontic steel wire was ligated around the first molar of the rat mandible for 4 weeks. Moreover, during this period, the rats were fed with 10% of sugar water and, if any ligature appeared to have loosened or fallen off, they were replaced immediately. These procedures were not conducted for the control group. Then, the orthodontic steel wires were removed, and the rats with periodontitis were left without treatment (the ligation group), while M-SAIB (approximately 50 mg) and MINO-M-SAIB (approximately 50 mg, 1 mg MINO) were injected into the periodontal pockets of rats with periodontitis immediately after the removal of the ligature or Periocline^®^ (approximately 8.33 mg, 0.17 mg MINO) was injected into the periodontal pockets once a week for 3 weeks and 6 weeks (*n* = 5, at each time point).

#### 2.10.2. Pharmacodynamic evaluation

In order to evaluate the periodontal status, the most important clinical periodontal parameters of gingival index (GI), the periodontal pocket depth (PD), were recorded, and the status of periodontal tissues around the maxillary first molar were also recorded via photograph. Five rats per group were checked at specific time points (0, 2, 4, and 6 weeks).

#### 2.10.3. Microcomputed tomography (micro-CT)

After the ligatures were removed, the rats were killed by administering an overdose of anesthetic (e.g. chloral hydrate) at 3 and 6 weeks. The specimens (the alveolar bone including the first, second, and third molars of rat maxillary) were collected and fixed in 4% paraformaldehyde for micro-CT scanning (Viva CT40; SCANCO Medical, Bruttisellen, Switzerland) at a resolution of 15 mm, energy of 70 kV, and power of 114 mA. Linear measurements of the alveolar bone loss (ABL) were taken from the cement-enamel junction (CEJ) to the alveolar bone crest (ABC) at the distal and mesial roots of the maxillary first molar in two-dimensional (2-D) micro-CT images (Park Chan et al., 2007). For the volumetric analysis, the bone volume/tissue volume (BV/TV) parameters were assessed in a 3-D region of interest (ROI) by using the mimics analysis software. In addition, the ROI axially involved a rectangle with the length and width encompassing the entire crown and vertically involving the height from the CEJ to the apex of the maxillary first molar. Then, the first molar was removed and the residual bone volume in the ROI was analyzed.

#### 2.10.4. Histology observation

The alveolar bone samples were decalcified with 10% ethylenediaminetetraacetic acid (EDTA) for 1.5 months at the room temperature. Then, the sample was dehydrated, embedded in paraffin, and sliced along the mesio-distal direction of the tooth to obtain a 5-μm-thick tooth-periodontal section. The sagittal section was then stained with hematoxylin and eosin (H&E) (Solabao, Beijing, China) to evaluate the pathological conditions of the periodontal tissues.

#### 2.10.5. Immunohistochemistry (IHI)

The expression of the osteoclastic marker RANK ligand (RANKL) and the osteogenic marker osteogenic growth peptide (OPG) was assessed at 3 and 6 weeks. The IHI-stained images were analyzed and the values of average optical density (AOD) of the images were measured for quantitative analysis by the ImagePro Plus 6.0 software.

### 2.11. Statistical analyses

All data were analyzed by the SPSS 20 software, and the data were expressed as the mean ± standard deviation. One-way analysis of variance (ANOVA), followed by Student–Newman–Keuls test, was employed to determine the statistical significance, with *p* < 0.05 considered to be statistically significant.

## 3. Results

### 3.1. Characteristics of MINO-microspheres and MINO-M-SAIB hybrid depots

In present study, the PLGA/PEG microspheres were prepared with different drug loading concentrations of MINO (0%, 10%, 12%, and 14% w/w relative to PLGA). For simplicity, the MINO-microspheres have been abbreviated as M (0%), M1 (10%), M2 (12%), and M3 (14%), respectively, in this study.

**Figure 1.**
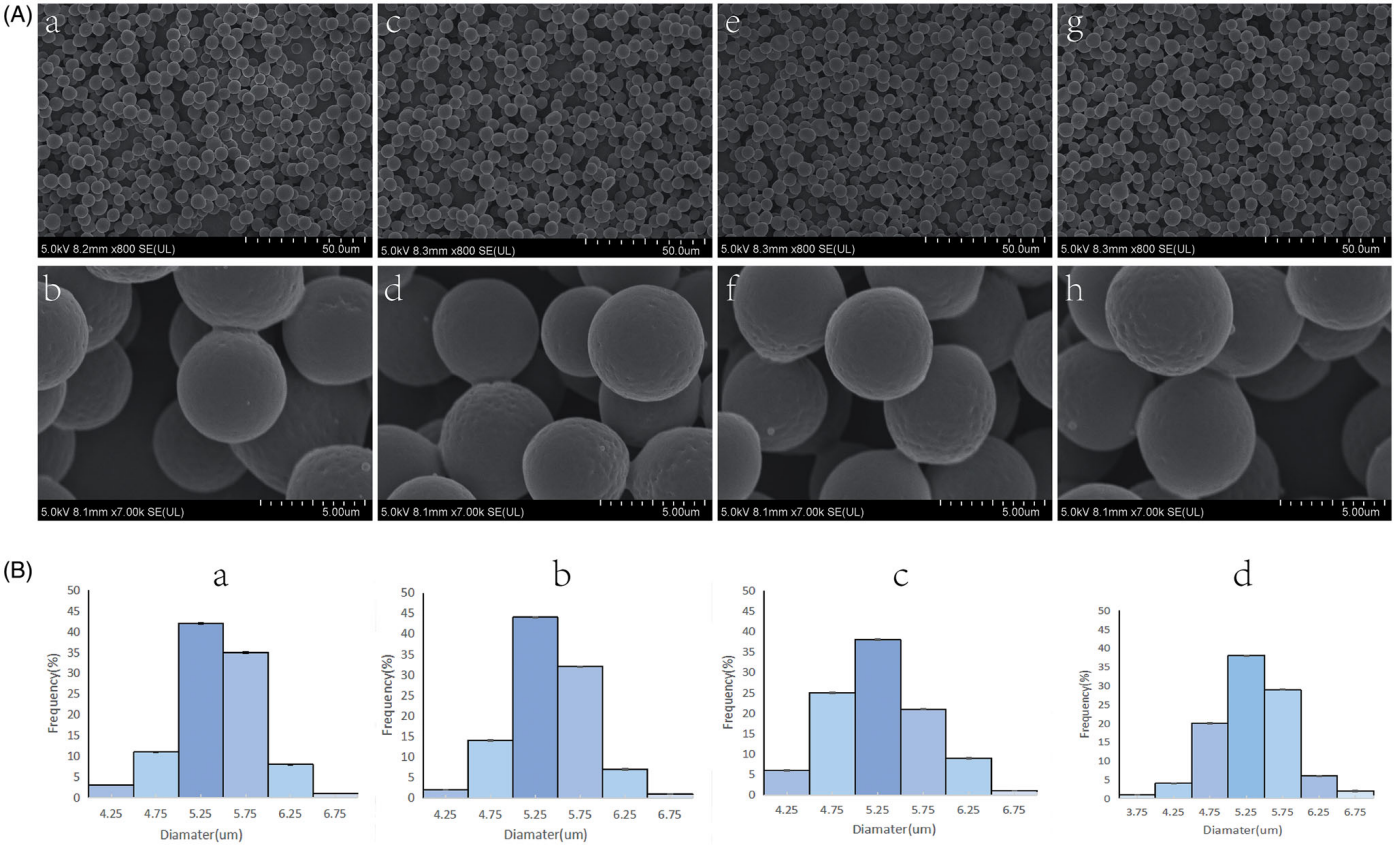
(A) SEM images of microspheres at 800 times (a, c, e, g) and 7000 times (b, d, f, h), including M (a, b), M1 (c, d), M2 (e, f), and M3(g, h). (B) Diameter distribution images of M (a), M1 (b), M2 (c), and M3 (d) (*n* = 100).

Figure 1(A) demonstrates the SEM images of the electrosprayed microspheres (including M, M1, M2, and M3). The MINO-microspheres were almost monodispersed and spherical in shape, and their surface morphology was slightly rough. The diameters distribution of MINO-microspheres are depicted in Figure 1(B), while Table 1 documents their diameter. The CV of M, M1, M2, and M3 were 8.43%, 7.88%, 9.72%, and 9.09%, and the diameter were 5.385 ± 0.454 µm, 5.429 ± 0.428 µm, 5.297 ± 0.515 µm, 5.354 ± 0.487 µm, respectively. Among the microspheres with different MINO loading capacities, the differences in the diameter were not statistically significant (*p* > .05).

**Table 1.** The characters of electrosprayed microspheres (mean ± SD, *n* ≥ 3).

	Diameter, *µm*	CV, %	Contact angle,°^a^	LE, %^a^	EE, %^a^
M	5.385 ± 0.454	8.43%	93.777 ± 0.303	–	–
M_1_	5.429 ± 0.428	7.88%	91.76 ± 0.1	8.71 ± 0.012	65.57 ± 3.07
M_2_	5.297 ± 0.515	9.72%	90 ± 0.25	11.148 ± 0.049	57.24 ± 1.45
M_3_	5.354 ± 0.487	9.09%	86.107 ± 0.487	12.64 ± 0.03	48.04 ± 3.24

^a^ All results showed statistically different.

Table 1 depicts that the drug loading of M1, M2, and M3 were 8.71 ± 0.012%, 11.148 ± 0.077%, and 12.64 ± 0.03%, which accounted for 87.01%, 92.89%, and 90.31% of theoretical drug loading, respectively. Furthermore, the encapsulation efficiency of M1, M2, and M3 were 65.57 ± 3.07%, 57.24 ± 1.45%, and 48.04 ± 3.24%, which demonstrated that the encapsulation efficiency decreased with increasing MINO loading capacity in the microspheres (*p* < .05).

**Figure 2.**
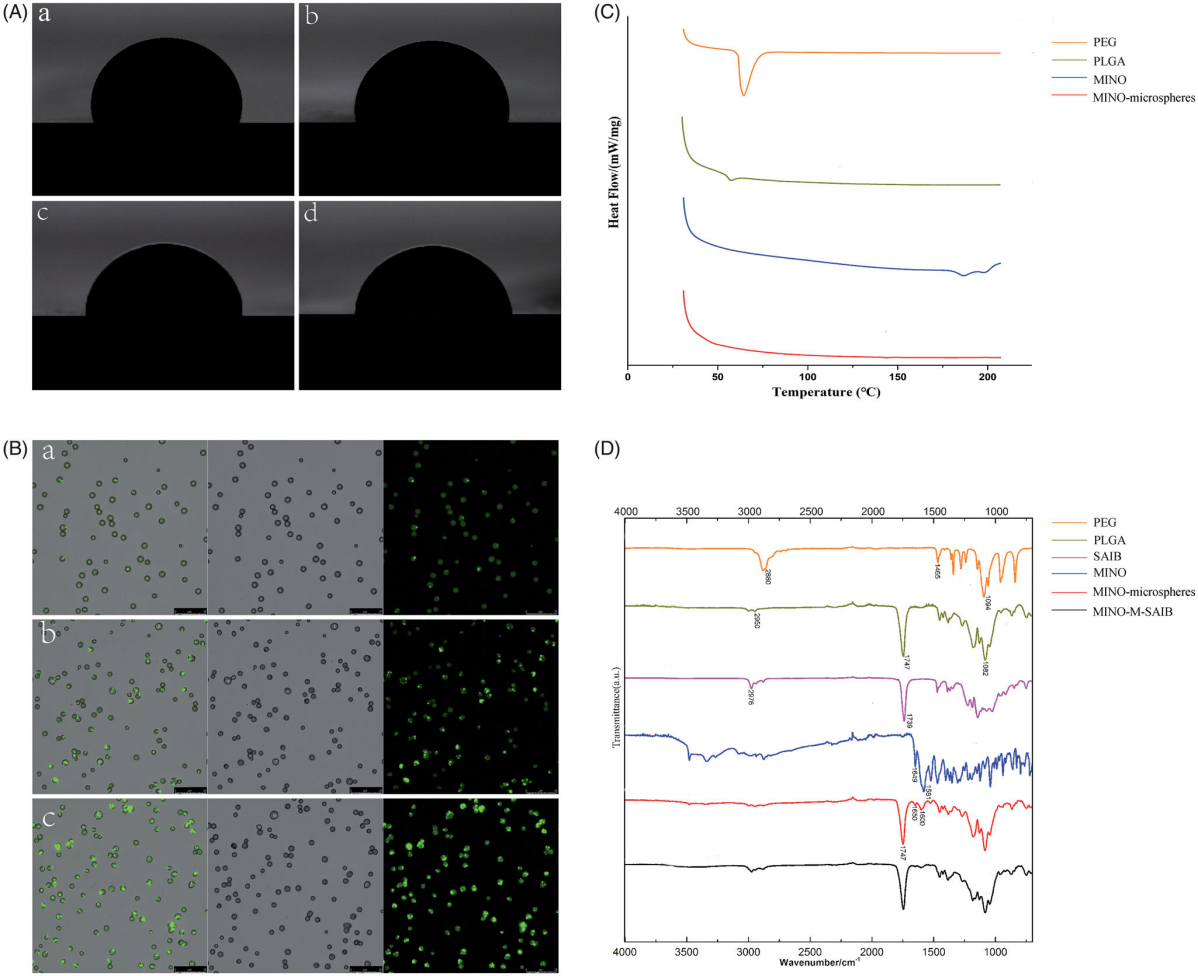
(A) Contact angle of microspheres of M (a), M1 (b), M2 (c), M3 (d). (B) Laser scanning confocal microscopy images of M1 (a), M2 (b), M3 (c). Composite picture, bright field picture and fluorescent picture from left to right, respectively (scale bar= 25 μ*m*). (C) DSC thermograms of PEG, PLGA, MINO, MINO-microspheres. (D) FTIR spectra of PEG, PLGA, SAIB, MINO, MINO-microspheres, MINO-M-SAIB.

Following the decrease in the value of contact angle, the material becomes more hydrophilic. As demonstrated in Figure 2(A), with increasing the amount of MINO in the microspheres, the contact angle keeps reducing with the contact angle of M0, M1, M2, and M3 as 93.777 ± 0.303°, 91.76 ± 0.1°, 90 ± 0.25°, and 86.107 ± 0.487°, respectively (Table 1). These results illustrate that the hydrophilicity of the micospheres increased with increasing concentration of MINO (*p* < .05).

Laser confocal microscopy was employed to visualize the distribution of MINO in microspheres. In Figure 2(B), the microspheres with different drug loading capacities are presented with circular yellow-green signals, depicting a relatively uniform distribution of MINO. In addition, when the theoretical drug loading of the electrosprayed microspheres increased from 12 to 14%, the fluorescence density of the microspheres began to increase, and stronger fluorescence density was concentrated on microspheres surface, which represented that the amount of MINO in the microspheres and the amount of MINO on the microspheres surface increased as the MINO loading.

DSC analysis was performed on MINO-loaded microspheres as well as MINO, PLGA and PEG (Figure 2(C)). The DSC curve for PLGA showed a small endothermic peak at 57.31 °C and PEG showed an endothermic peak at 64.4 °C for its melting. The MINO thermal analysis revealed two endothermic peaks at 186.48 °C and 197 °C, approximately followed by its degradation. Moreover, no obvious endothermic peak was observed in the MINO-loaded microspheres, which indicated that the thermal stability of the microspheres is improved when compared with the raw materials. Moreover, the MINO peaks were not visualized in the MINO-loaded microspheres indicating its change from the crystalline form to the amorphous form.

The FTIR spectra of MINO-microspheres, MINO-M-SAIB hybrid depot, and their constitution are demonstrated in Figure 2(D). The absorption bands of PEG emerged at 2880 cm^−1^ and 1465 cm^−1^, which can be a result of –CH2 stretching and bending vibrations, respectively, while the C–O–C stretching vibrations (1094 cm^−1^) were observed in PEG (Ebadi et al., 2019). The bands of –CH2 and –CH3 stretching vibrations (2950 cm^−1^), the –COOH stretching vibrations (1747 cm^−1^), and the C–O–C stretching vibrations (1082 cm^−1^) were demonstrated in the spectra of PLGA, which agreed with previously published data (Fu et al., 2017). The two peaks of –CH3 stretching vibrations (2976 cm^−1^) and C = O stretching vibrations (1739 cm^−1^) were detected in SAIB. The characteristic peaks at 1649 cm^−1^ and 1581 cm^−1^, due to the stretching vibrations of C = C on the benzene ring and the skeleton vibrations of benzene ring respectively, were testified in the spectra of MINO. In addition, the spectra of MINO exhibited multiple complex absorption peaks in the range of 500–1400 cm^−1^, as a consequence of the four benzene rings in the molecular structure of MINO. The absorption peaks at 1747 cm^−1^, 1650 cm^−1^ and 1600 cm^−1^, owing to the –COOH stretching vibrations of PLGA and the benzene ring vibrations in MINO, were detected in the spectra of MINO-microspheres, which manifested that the MINO was encapsulated into the microspheres. Nevertheless, the characteristic peaks of PEG were not clearly displayed in the spectra of microspheres, which may be involved with the fact that the main absorption peaks of PEG partially coincide with those of PLGA and MINO. Furthermore, compared with the MINO-microspheres, there was no new absorption peak in the spectra of MINO-M-SAIB, which demonstrated that the combination mode of SAIB and MINO-microspheres belonged to physical blending.

### 3.2. *In vitro* release of MINO-microspheres and MINO-M-SAIB hybrid depots

**Figure 3.**
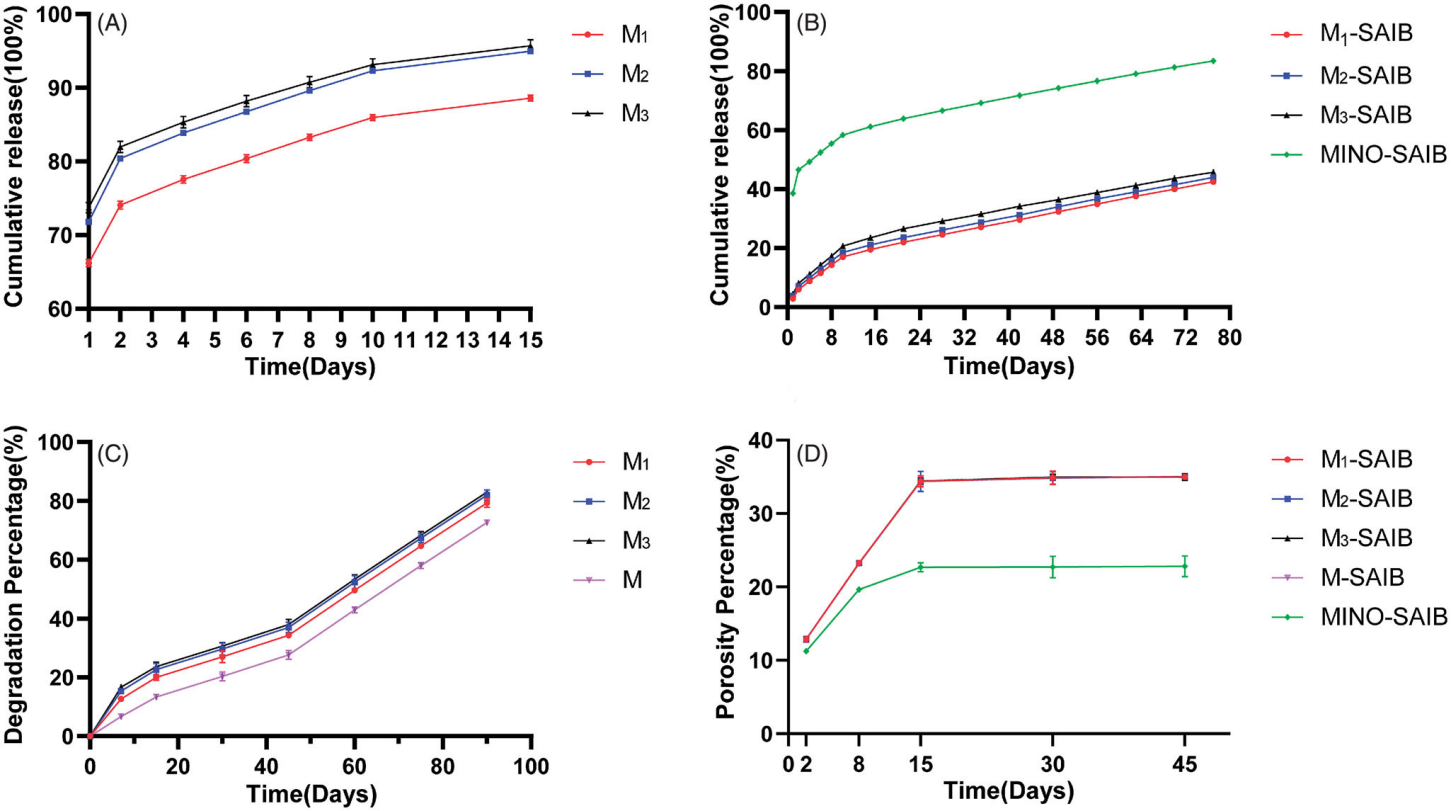
(A) In vitro release from MINO-microspheres. (B) In vitro release from depots. (C) In vitro degradation of MINO-microspheres. (D) The porosity of depots. The results represent the average ± SD (*n* = 3 for release, degradation and porosity).

Figure 3(A) depicts the release curves of MINO-microspheres (M1, M2, and M3). On the first day, a serious burst release (>65%) was observed in all MINO-microspheres. The cumulative release from M1 was >75%, and the amount of release from M2 and M3 was >80% after 4 days, after which the release patterns of MINO-microspheres were featured by a steady release rate (approximately 2.7% every day) until the 15th day. Finally, the amount of cumulative release was nearly 90% from all groups on the 15th day.

The *in vitro* release profiles from MINO-M-SAIB and MINO-SAIB depots are exhibited in Figure 3(B). After the MINO-microspheres (M1, M2, and M3) were dispersed into the SAIB solution to form hybrid depots, the initial burst release decreased significantly from 66.18 to 2.92%, from 71.82 to 3.82%, and from 73.75 to 4.45% on the first day, respectively. Nevertheless, an initial burst release (of up to 38.63%) continued to be displayed in MINO-SAIB depot. Over the first 10 days, the MINO-SAIB and MINO-M-SAIB (i.e. M1-SAIB, M2-SAIB, and M3-SAIB) depots demonstrated fast drug release rate with a cumulative release rate of 58.3%, 17.06%, 18.57%, and 20.7% on the 10th day, respectively. After 10 days, the release profiles of the depots were all featured by a sustained rate (of >0.38% per day) until the 77th day.

Some mathematical models have been found to be acceptable for the analysis of drug release, such as the zero order (equation: Q = a + K_0_t), first-order (equation: Q = a(1 − e^−k^_1_^t^)), Higuchi (equation: Q = a + K_H_t^1/2^), and Ritger-Peppas (equation: Q = K_R_t^n^) models (Ritger & Peppas, 1987; Cai et al., 2011; Haroosh et al., 2014). In our study, the experimental data of drug release were fitted by these four kinetic models to better understand the release mechanism; Table 2 presents the obtained model parameters. The Ritger-Peppas equation showed high R^2^ value (R^2^ > .99) to all kinetic data, which represented the best correlation with the release data. Therefore, the Ritger-Peppas equation was applied to analyze the MINO release from depots, the acceptable regression coefficients and the slopes, and the degree of correlation and drug release rate of different depots, respectively, are all represented in Table 3. Meanwhile, the linear fits of the MINO release profiles revealed the existence of two release stages for the depots.

**Table 2.** Parameters obtained by fitting four different models to the release data for SAIB-based depots drug release kinetics (mean ± SD).

Model Type	M1-SAIB	M2-SAIB	M3-SAIB	MINO-SAIB
Zero-order model	K_0_=0.46559 ± 0.02891	K_0_=0.47088 ± 0.03081	K_0_=0.48221 ± 0.0368	K_0_=0.48879 ± 0.04165
	b = 9.0201 ± 1.14256	B = 10.29659 ± 1.21789	b = 12.00746 ± 1.45445	b = 49.37839 ± 1.6462
	R^2^=.945	R^2^=.939	R^2^=.919	R^2^=.901
First-order model	K_1_=0.0407 ± 0.00587	K_1_=0.04608 ± 0.00667	K_1_=0.05472 ± 0.00704	K_1_=0.40153 ± 0.09959
	b = 40.12381 ± 2.2249	b = 40.55362 ± 2.10357	b = 41.5458 ± 1.75451	b = 70.00304 ± 2.86447
	R^2^=.949	R^2^=.941	R^2^=.945	R^2^=.479
Higuchi model	K_H_=4.77558 ± 0.10119	K_H_=4.8414 ± 0.10948	K_H_=5.00013 ± 0.13618	K_H_=5.08988 ± 0.19476
	b= −0.25675 ± 0.55825	b = 0.85752 ± 0.604	b = 2.13393 ± 0.7513	b = 39.26448 ± 1.0745
	R^2^=.993	R^2^=.992	R^2^=.988	R^2^=.978
Ritger-Peppas model	K_R_=4.72755 ± 0.2694	K_R_=5.53374 ± 0.29036	K_R_=6.68178 ± 0.33467	K_R_=39.0289 ± 0.68128
	*n* = 0.50037 ± 0.01474	*n* = 0.47212 ± 0.01365	*n* = 0.44087 ± 0.01312	*n* = 0.16815 ± 0.00502
	R^2^=.993	R^2^=.993	R^2^=.992	R^2^=.989

**Table 3.** Evaluation of drug release kinetics of SAIB-based depot according to the Ritger-Peppas equation.

Depot Stage, d	M1-SAIB		M2-SAIB		M3-SAIB		MINO-SAIB	
	1–10d	10–77d	1–10d	10–77d	1–10d	10–77d	1–10d	10–77d
Slope^a^	1.42998 ± 0.00588	0.37367 ± 0.00135	1.49536 ± 0.00615	0.37978 ± 0.00138	1.62893 ± 0.0067	0.35531 ± 0.00133	1.7763 ± 0.01286	0.34716 ± 0.0022
R^2^	0.991	0.987	0.991	0.989	0.989	0.989	0.966	0.974

^a^ The slope represented the drug release rate according to the Ritger-Peppas equation (mean ± SD).

### 3.3. *In vitro* degradation of MINO-microspheres

As shown in Figure 3(C), the degradation behavior of MINO-microspheres (i.e. M, M1, M2, and M3) were reported, and the linear fits of the MINO-microspheres degradation profiles demonstrated that M involved 2 degradation stages, while M1, M2, and M3 involved three degradation stages (Table 4). According to pseudo-first-order kinetics (Siepmann et al., 2005), the degradation curves of the microspheres were good-fitted, which was reflected by the acceptable regression coefficients, and the slope represented the microspheres degradation rate (Table 4). In addition, during the first 7 days, M3, M2, and M1 exhibited faster degradation rate than M, which can mainly be attributed to the drug release amount from the MINO-microspheres. From days 7 to 90, the degradation rates of all microspheres (concluding M, M1, M2, and M3) were almost the same and the amount of degradation was approximately 66.67%, indicating that the concentration of MINO was irrelevant to the degradation of the microspheres (*p* > .05). Notably, the loss of weight of microspheres was accelerated after 45 days. Finally, the degradation amount of all microspheres (including M, M1, M2, and M3) were 79.3%, 82%, 83%, and 72.7% until the 90th day, respectively.

**Table 4.** Evaluation of degradation kinetics of microspheres according to pseudo-first order equation.

	M1			M2			M3			M	
Degradation Stage	0–7d	7–45d	45–90d	0–7d	7–45d	45–90d	0–7d	7–45d	45–90d	0–45d	45–90d
Slope^a^	0.17931 ± 6.89402E-4	0.05183 ± 4.48809E-4	0.09844 ± 1.32837E-4	0.21599 ± 0.00106	0.05182 ± 5.42725E-4	0.09873 ± 9.6929E-5	0.233 ± 0.00133	0.05172 ± 5.89863E-4	0.09901 ± 5.08543E-18	0.05928 ± 1.27293E-18	0.09783 ± 2.29681E-4
R^2^	1	0.985	0.999	1	0.981	0.999	1	0.977	0.999	0.995	0.997

^a^ The slope represented the degradation release rate according to the pseudo-first order equation (mean ± SD).

### 3.4. The porosity of depots

The porosity profiles of different depots are demonstrated in Figure 3(D). From days 2 to 45, the porosity of the MINO-M-SAIB hybrid depots (including M1-SAIB, M2-SAIB, M3-SAIB, and M-SAIB) was nearly consistent (*p* > .05), but always higher than that of the MINO-SAIB depot. Moreover, the change rates of porosity of the MINO-M-SAIB hybrid depots were greater than that of the MINO-SAIB depot at all time points. The porosity of all groups increased at a quick rate in the first 15 days, but remained steady from days 15 to 45.

### 3.5. Cytotoxicity of the drug delivery systems on osteoblastic cells

**Figure 4.**
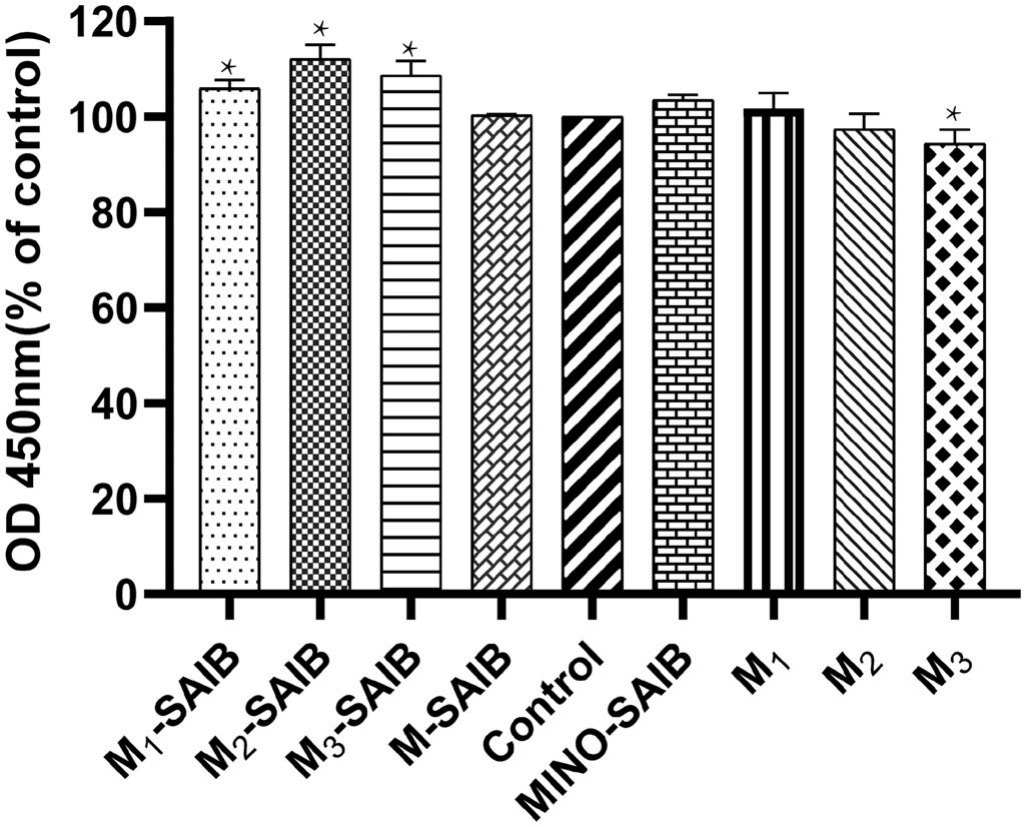
CCK-8 assays of cells cultured with the extracts of MINO-microspheres and the depots. The data represented the mean ± SD, *n* = 3, *p < .05.

The cytotoxicity of different extracts from depots and MINO-microspheres was analyzed by the CCK-8 assay. As shown in Figure 4, when compared with the control, the five extracts of M1-SAIB, M2-SAIB, M3-SAIB, MINO-SAIB, and M1 were found to promote the proliferation of osteoblasts to a greater extent, while the M2 and M3 groups demonstrated a slight cytotoxicity. Moreover, no significant difference was evident between the M-SAIB and control groups. Interestingly, the differences between these groups (including M1-SAIB, M2-SAIB, M3-SAIB, and M3) and the control group were statistically significant. Generally, the results revealed that minocycline could promote the proliferation of osteoblasts in a certain concentration range, while, on the contrary, a high concentration of minocycline could inhibit the proliferation of osteoblasts. These findings cumulatively suggest that M2-SAIB mostly potentiate osteoblast cell growth; hence, M2-SAIB was used in animal experiments in the present research.

### 3.6. *In vivo* studies

#### 3.6.1. Micro-CT findings

**Figure 5.**
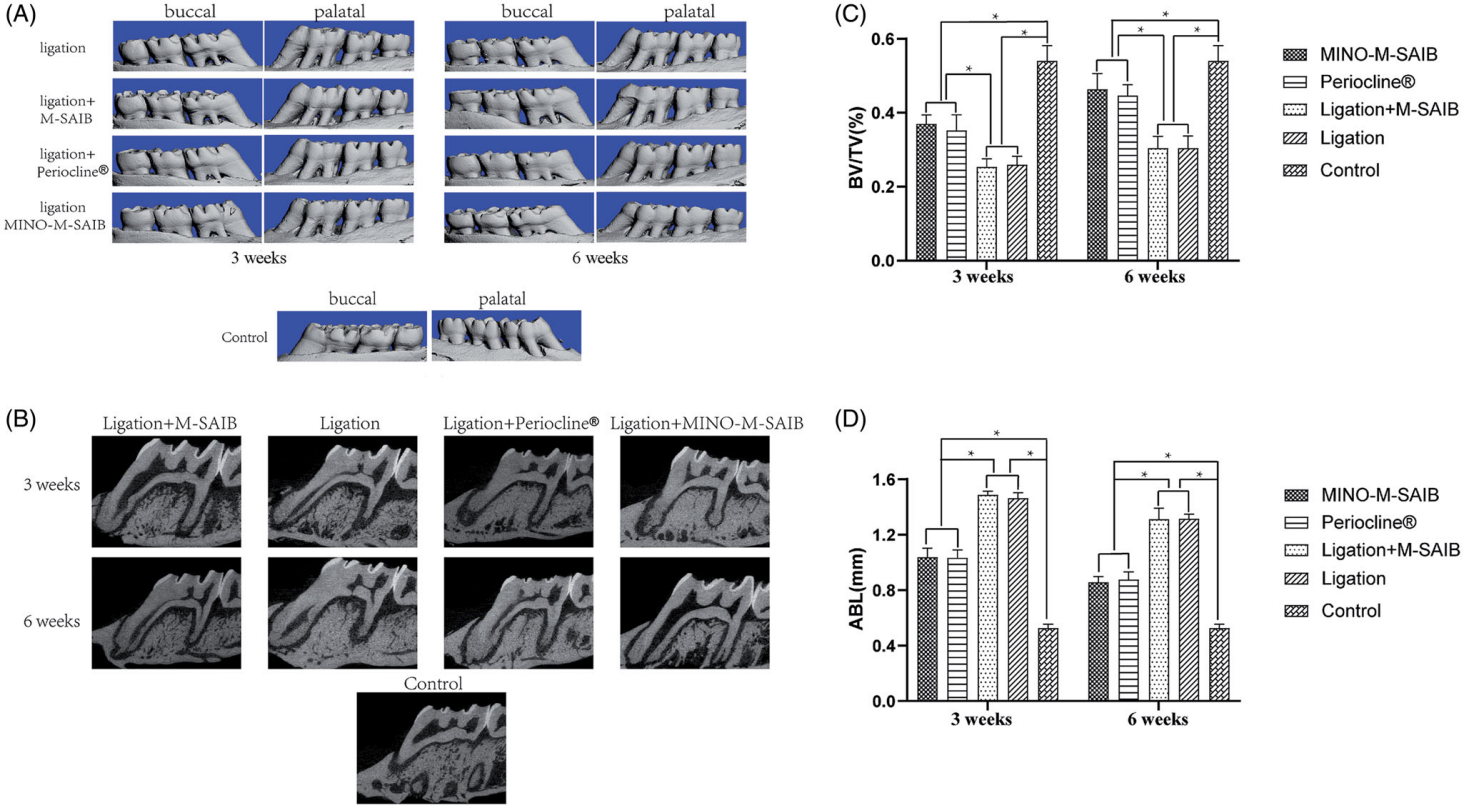
(A) Three-dimensional micro-CT images (buccal and palatal view) of the maxilla first molars alveolar bone level in different groups at 3 weeks and 6 weeks. (B) Bi-dimensional(2D) images of the maxilla first molars alveolar bone level in different groups at 3 weeks and 6 weeks. (C) Analysis of micro-CT volumetric parameters: bone volume/tissue volume (BV/TV) at 3 weeks and 6 weeks. (D) Analysis of micro-ct linear bone loss: alveolar bone loss (ABL) at 3 weeks and 6 weeks. The data represented the mean ± SD, *n* = 5, *p < .05.

When compared to the ligation and ligation + M-SAIB groups, an obvious increase was noted in the alveolar crest height in the ligation + MINO-M-SAIB and Periocline^®^ groups at 3 and 6 weeks, as reflected in the 2-D and 3-D micro-CT images of maxillary first molar (Figure 5(A,B)). As presented in the study (Figure 5(C,D)), the results of volumetric bone loss and linear bone loss, as reflected by BV/TV and ABL, all demonstrated a significant preventive effect on the bone loss caused by periodontitis for the ligation + MINO-M-SAIB and Periocline^®^ groups at 3 and 6 weeks when compared with the ligation and ligation + M-SAIB groups (*p* < .05). In addition, in the ligation, ligation + M-SAIB, ligation + MINO-M-SAIB, and Periocline^®^ groups, the respective ABL values were 1.464 ± 0.035 mm, 1.489 ± 0.024 mm, 1.038 ± 0.058 mm, and 1.033 ± 0.05 mm, respectively, at 3 weeks, and 1.316 ± 0.03 mm, 1.313 ± 0.071 mm, 0.858 ± 0.035 mm, and 0.876 ± 0.05 mm, at 6 weeks, while the ABL value in the control group was 0.527 ± 0.025 mm. At 3 and 6 weeks, when compared to that of the Periocline^®^ group, a slightly greater improvement was noted in the volumetric bone loss and linear bone loss in the MINO-M-SAIB group (*p* > .05). Cumulatively, the MINO-M-SAIB hybrid depot showed a significant preventive effect in bone loss in the periodontitis patients.

#### 3.6.2. Pharmacodynamic outcomes

**Figure 6.**
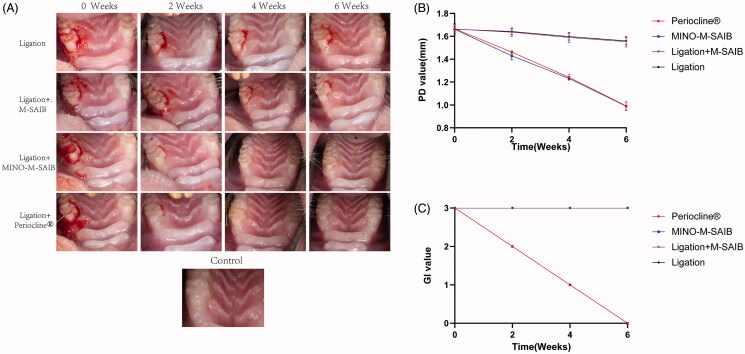
(A) The photographs of periodontal tissue in different groups at 0, 2, 4, 6 weeks. (B) The values of PD for the maxillary first molar in different groups. (C) The values of GI for the maxillary first molar in different groups. The data represented the mean ± SD, *n* = 5.

As is exhibited in Figure 6(A), at the baseline (0 week), the redness, bleeding, and swelling of the gingival area around the maxillary first molar were obviously noted in all groups; over a period of time, gingival swelling and bleeding were significantly improved in the MINO-M-SAIB and Periocline^®^ groups, although no significant improvement was noted in the ligation and ligation-M-SAIB groups. The values of GI and PD for the maxillary first molar at different observation time points are listed in Figure 6(B,C). From 0 to 6 weeks, the PD values of all groups decreased over a period of time and were significantly different from those of the control group (*p* < .05); however, the MINO-M-SAIB and Periocline^®^ groups indicated lower PD value than the ligation and ligation + M-SAIB groups at all time points (*p* < .05). Moreover, the GI value in the MINO-M-SAIB and Periocline^®^ groups decreased gradually during 0–6 weeks, albeit it remained high in the ligation and ligation + M-SAIB groups. Based on the quantification of clinical periodontal parameters, the MINO-M-SAIB hybrid depot demonstrated a good anti-inflammatory efficiency on the animal model of ligature-induced periodontitis.

#### 3.6.3. Histological observations

**Figure 7.**
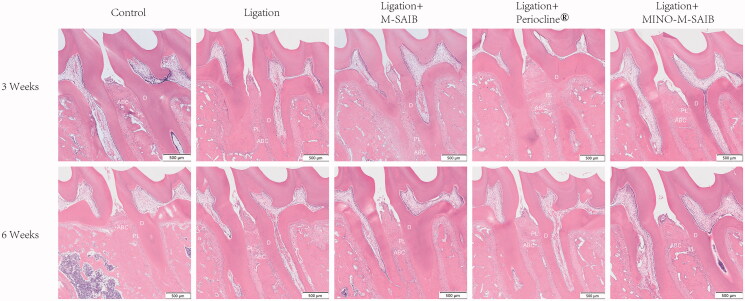
Histological observation (H&E stain) of maxillary periodontal tissue after 3 and 6 weeks (40 × magnification, bar= 500 μm). ABC: Alveolar bone crest; PL: Periodontal ligament; D: Dentin.

From the H&E stained sections (Figure 7), when compared to the normal periodontal tissues around the maxillary first molar in the control group, an obvious periodontal pocket created as a result of the proliferation of epithelial root, obvious inflammatory infiltration, and a significantly absorbed alveolar bone were noted in the ligation and ligation + M-SAIB groups. On the contrary, in the Periocline^®^ and ligation + MINO-M-SAIB groups, the gingival junction epithelium was re-attached to CEJ and a significantly increased alveolar bone height were recorded at 3 and 6 weeks.

#### 3.6.4. IHI analyses

**Figure 8.**
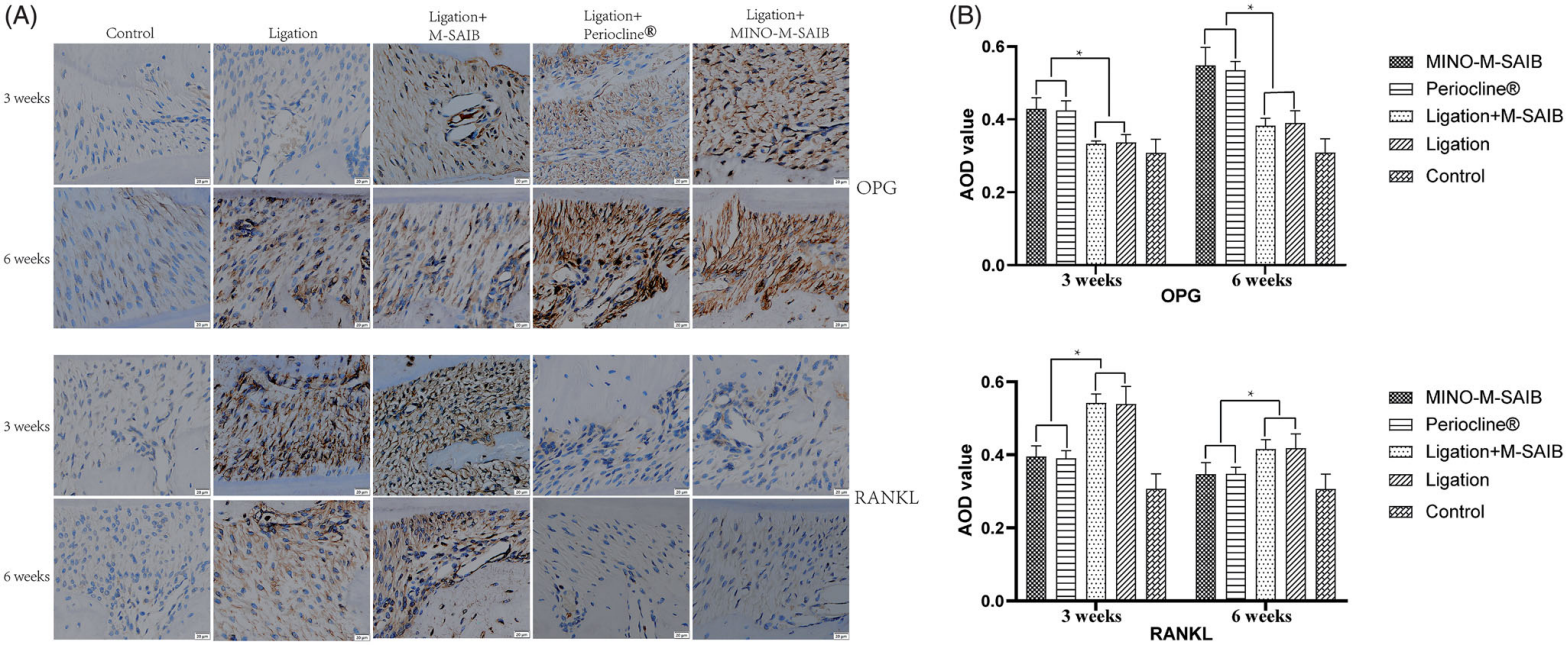
IHC staining and the corresponding quantitative analysis of OPG and RANKL after 3 and 6 weeks (400 × magnification, bar = 20 μm). The results represent the mean ± SD, *n* = 3, *p < .05.

As shown in Figure (8(A)), when compared to the ligation and ligation + SAIB groups, the expression of OPG protein significantly increased and the expression of RANKL significantly decreased at 3 and 6 weeks in the Periocline^®^ and ligation + MINO-M-SAIB groups, while the expressions of OPG and RANKL in the control group were the lowest among all compared groups. In addition, this difference was also evident in the quantitative analysis of the expressions of OPG and RANKL (Figure 8(B)).

## 4. Discussion

For the treatment of periodontitis, the success of the local application of MINO mainly depends on its sustained release and appropriate concentration in the periodontal pockets (Gibson et al., 2019). Consequently, the present study mainly focused on the preparation of the MINO-M-SAIB hybrid depot that can ensure both a sustained release and an appropriate concentration of the antibiotic. The MINO-M-SAIB hybrid depot was expected to achieve antibacterial activity while also promoting new bone formation.

For the microspheres, the composition of the MINO-M-SAIB hybrid depot, their surface morphology was slightly rough, which may be because the solvent did not totally evaporate before falling down to the aluminum foil during electrospray (Yao et al., 2016). Given that particles with a rough surface enhance cell adhesion to facilitate cellular internalization (Chen et al., 2018), the surface morphology of the microspheres in our study was favorable to the attachment of osteoblasts to the microparticles. Furthermore, the electrosprayed microspheres had a narrow size distribution and relatively uniform drug distribution, consistent with Yao^′^s study (Yao et al., 2016). With increase in the theoretical drug loading, the actual drug loading of the electrosprayed microspheres increased, which achieved an agreement with previous studies (Yang et al., 2019). However, the ratio of actual drug loading to theoretical drug loading in the electrosprayed microspheres began to decrease when the theoretical drug loading increased from 12 to 14%, which was probably because of the limited ability of PLGA microspheres to carry drugs. Moreover, for the microspheres with different drug loading, both the results of encapsulation efficiency and the laser confocal images of the drug distribution showed that the amount of MINO on the microsphere surface gradually increased with the increase in the drug loading of the microspheres. The results could be interpreted by the fact that the increase in the concentration of MINO would make it difficult for dissolved MINO to draw toward the center of the droplet as the solvent evaporated; therefore, MINO deposited on the microsphere surface (Hong et al., 2008).

In the in vitro drug release experiment, the electrosprayed microspheres showed an obvious burst release (up to 65%); however, after the MINO-microspheres were loaded into SAIB, the burst release of MINO significantly decreased, which was in agreement with the findings of several studies (Lee et al., 2006; Wang et al., 2016; Yang et al., 2019). For the release behavior of depots, at the early stage from 1 to 10 days, most of the drug concentration in the MINO-M-SAIB hybrid depots was contained in the microspheres; thus, only a small amount of MINO on the microsphere surface may be released into SAIB, resulting in a significant decrease in burst release; meanwhile, an obvious burst release was observed in the MINO-SAIB depot because of a large amount of the drug dissolved in the depot. From 10 to 77 days, the release rate of MINO-SAIB depot was lower than that of the MINO-M-SAIB hybrid depots (Table 3), which could be mainly attributed to the fewer concentration of the drug left in the MINO-SAIB hybrid depot in the latter stage. Notably, with the decrease in EE of the microspheres (i.e. increase in the amount of MINO on the microsphere surface), the amount of drug burst release in the MINO-M-SAIB hybrid depots increased, which could be related to the increase in the amount of dissolved drug in SAIB from the microsphere surface (Lin et al., 2015).

Considering the release behavior of microspheres in the first 7 days, during which all MINO-microspheres released up to 80%, it can be considered that the amount of microsphere degradation was mainly attributed to the release loss of MINO. At the same time, during the first 7 days, the degradation rates of M1, M2, and M3 were higher than those of the blank microsphere M, which was also because of the release amount of MINO-microspheres. As shown in previous studies, the hydrolytic mechanism is the mechanism by which PLGA microspheres degrade, and during degradation, at the early stage, the increase in surface roughness and surface defects could be observed on the microspheres with gradual penetration of water; correspondingly, at the latter stage, increasing large cavities were formed by water penetration expanded, which induced faster degradation of the microspheres (James et al., 2012; Xu et al., 2013). This phenomenon corroborates with the results of our research, indicating that the degradation rate of the microspheres after 45 days was greater than that before 45 days. Additionally, it is worth noting that the porosity of the depots was may be relevant to their release behavior: from 2 to 15 days, the porosity increased rapidly, which corresponded to the rapid drug release in the depots, whereas after 15 days, the porosity increased only slowly, corresponding to the sustained and steady drug release. Notably, although the porosity of the MINO-SAIB depot was lower than that of the MINO-M-SAIB hybrid depots, the drug release rate of the former was greater in the first 10 days, mainly due to more amount of MINO dissolved in the MINO-SAIB depot, thereby verifying that the release behavior of depots is mainly controlled by the amount of drug dissolved in it.

Several studies have demonstrated that MINO in a therapeutic concentration range can potentiate osteoblast cell growth and that with a high concentration will inhibit the proliferation of osteoblasts (Almazin et al., 2009; Calasans-Maia et al., 2019), and combining the results of the CCK-8 assay with the release behavior of depots and microspheres, the results of the present study also confirm this point. In addition, the minimum inhibitory concentrations of MINO against *P. actinomycetemcomitans, Porphyromonas gingivalis*, and *Treponema* were only 0.25 µg/ml, 0.125 µg/ml, and 0.125 µg/ml, respectively (Andrés et al., 1998; Naoko et al., 2007; Okamoto-Shibayama et al., 2017). Therefore, the release behavior of MINO in the present study, which was featured by an initial burst release (about 3%) and a sustained rate (over 0.38% per day) until the 77th day, could not only achieve an effective antibacterial concentration of MINO but also ensure osseointegration.

In our in vivo research, the MINO-M-SAIB hybrid depot showed an obvious improved efficiency on ligature-induced periodontitis, as reflected in the results of micro-CT and pharmacodynamic evaluation, which mainly was attributed to the osteogenic and antibacterial ability of MINO and the sustained release of MINO from the MINO-M-SAIB hybrid depot. In addition, at 3 and 6 weeks, there was no significant difference in the results of pharmacodynamic evaluation, bone volume, and bone height between the ligation and ligation + M-SAIB groups, indicating that M-SAIB alone neither damaged the periodontal tissues nor stimulated bone formation. Periocline^®^, a bio-absorbable sustained local drug delivery system containing 20 mg/g MINO, has been widely used clinically recently (Yang et al., 2018); however, the release behavior of MINO from the system exists a marked initial burst release (up to 40%) and only lasts for 7 days (Wang et al., 2012). As a result, Periocline^®^ is usually administered by injection into the periodontal pocket once a week, and repeated requests for multiple visits may cause inconvenience to patients and lead to poor compliance with treatment procedures. Moreover, some previous studies have shown that Periocline^®^ is not conducive to osteogenesis due to large fluctuations of the drug concentration in the periodontal pocket (Vandekerckhove et al., 1998; Liu et al., 2013). However, in our study, MINO-M-SAIB, a carrier with a sustained and long-time release of MINO, achieved the same treatment effect of periodontal inflammation as Periocline^®^, as well as a slightly greater osteogenesis effect than Periocline^®^, as reflected by the results of micro-CT and pharmacodynamics evaluation at 6 weeks. Finally, it is worth noting that in our previous study, the 2% MINO-PLGA membrane was still characterized by an initial burst release of 20% and its osteogenesis effect against periodontitis was studied at 3 and 6 weeks (Ma et al., 2020); correspondingly, the therapeutic outcomes at 3 and 6 weeks was also evaluated in the present study for comparison with our previous study. The results showed that both MINO-M-SAIB and MINO-PLGA membrane achieved good osteogenic effects, and a better sustained release behavior was observed with the MINO-M-SAIB hybrid depot, while also being injectable, so that it will be easier to place the depots in the periodontal pockets than membranes.

The formation of new bone is mainly controlled by osteoblasts and osteoclasts by regulating the expression of RANKL and its decoy receptor, osteoprotegerin (OPG) (Xu et al., 2014). RANKL binds to its receptor, RANK, which is expressed by osteoclast precursor cells, in order to trigger precursors to differentiate into osteoclasts (Harada & Takahashi, 2011). Moreover, OPG, which is expressed by osteoblasts, blocks osteoclast activation due to its high-affinity binding to RANKL (Wang et al., 2020). Therefore, combined with the present results about the expression of OPG and RANKL, it can be suggested that MINO promotes the formation of new bone by downregulating RANKL and upregulating OPG.

## 5. Conclusions

In the present study, the MINO-M-SAIB hybrid depots were successfully prepared and showed a significantly improved controlled release performance of MINO and good anti-inflammatory and osteogenic effects in ligature-induced periodontitis in SD rats. Moreover, the findings indicate that the use of the MINO-M-SAIB hybrid depot can decrease the numbers of patient visits and can be easily placed in the periodontal pocket, as a result of its sustained release pattern and injectability. Taken together, the present study suggests that the MINO-M-SAIB hybrid depot has great potential for clinical application in the treatment of periodontitis.
